# 6-Isopropyl-3-phenyl-5-(*p*-tol­yloxy)-3*H*-1,2,3-triazolo[4,5-*d*]pyrimidin-7(6*H*)-one: whole-mol­ecule disorder

**DOI:** 10.1107/S1600536809039798

**Published:** 2009-10-07

**Authors:** Xiao-Hua Zeng, Shou-Heng Deng, Ping Chen, Hong-Mei Wang, Hai-Tao Gao

**Affiliations:** a Institute of Medicinal Chemistry, Yunyang Medical College, Shiyan 442000, People’s Republic of China; b Center of Oncology, People’s Hospital affiliated with Yunyang Medical College, Shiyan 442000, People’s Republic of China; c Department of Medicinal Chemistry, Yunyang Medical College, Shiyan 442000, People’s Republic of China

## Abstract

The title compound, C_20_H_19_N_5_O_2_, exhibits whole-mol­ecule disorder the refined ratios of the two components being 0.57 (2):0.43 (2). In the major component, the essentially planar [maximum deviation 0.033 (17) Å] fused pyrimidine and triazole ring system forms a dihedral angle of 10.5 (3)° with the phenyl ring, while in the minor component of disorder this angle is 27.5 (5)°. The crystal structure is stabilized by π–π stacking inter­actions between symmetry-related triazole and pyrimidine rings, with centroid–centroid distances of 3.594 (10) Å.

## Related literature

For the biological activity of 8-aza­guanine derivatives see: Roblin *et al.* (1945 ▶); Ding *et al.* (2004 ▶); Mitchell *et al.* (1950 ▶); Levine *et al.* (1963 ▶); Montgomery *et al.* (1962 ▶)); Yamamoto *et al.* (1967 ▶); Bariana (1971 ▶); Holland *et al.* (1975 ▶). For related structures, see: Ferguson *et al.* (1998 ▶); Zhao, Xie *et al.* (2005 ▶); Zhao, Hu *et al.* (2005 ▶); Zhao, Wang & Ding (2005 ▶); Chen & Shi (2006 ▶); Maldonado *et al.* (2006 ▶); Xiao *et al.* (2007 ▶); Wang *et al.* (2006 ▶, 2008 ▶); Zeng, Deng *et al.* (2009 ▶), Zeng, Liu *et al.* (2009 ▶). For examples of whole-mol­ecule disorder, see: Kirsop *et al.* (2006 ▶); Cox & Wardell (2003 ▶). For the preparation, see: Zeng *et al.* (2006 ▶).
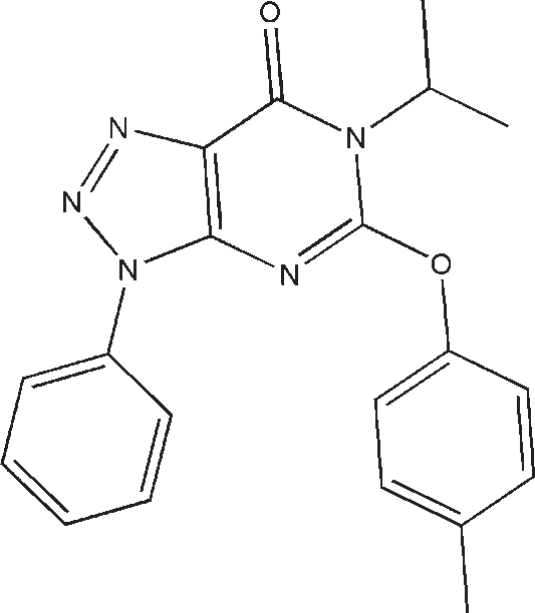


## Experimental

### 

#### Crystal data

C_20_H_19_N_5_O_2_
*M* *_r_* = 361.40
Orthorhombic, 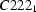

*a* = 10.2335 (6) Å
*b* = 21.8532 (12) Å
*c* = 16.7441 (9) Å
*V* = 3744.6 (4) Å^3^
*Z* = 8
Mo *K*α radiation
μ = 0.09 mm^−1^
*T* = 298 K
0.20 × 0.20 × 0.20 mm

#### Data collection

Bruker SMART CCD diffractometer
Absorption correction: none
21260 measured reflections
2285 independent reflections
1790 reflections with *I* > 2σ(*I*)
*R*_int_ = 0.023

#### Refinement

*R*[*F* ^2^ > 2σ(*F* ^2^)] = 0.050
*wR*(*F* ^2^) = 0.144
*S* = 1.06
2285 reflections
446 parameters
15 restraints
H-atom parameters constrained
Δρ_max_ = 0.17 e Å^−3^
Δρ_min_ = −0.17 e Å^−3^

### 

Data collection: *SMART* (Bruker, 2001 ▶); cell refinement: *SAINT* (Bruker, 2001 ▶); data reduction: *SAINT*; program(s) used to solve structure: *SHELXS97* (Sheldrick, 2008 ▶); program(s) used to refine structure: *SHELXL97* (Sheldrick, 2008 ▶); molecular graphics: *PLATON* (Spek, 2009 ▶); software used to prepare material for publication: *SHELXTL97* (Sheldrick, 2008 ▶).

## Supplementary Material

Crystal structure: contains datablocks global, I. DOI: 10.1107/S1600536809039798/lh2904sup1.cif

Structure factors: contains datablocks I. DOI: 10.1107/S1600536809039798/lh2904Isup2.hkl

Additional supplementary materials:  crystallographic information; 3D view; checkCIF report

## Figures and Tables

**Fig. 1.**
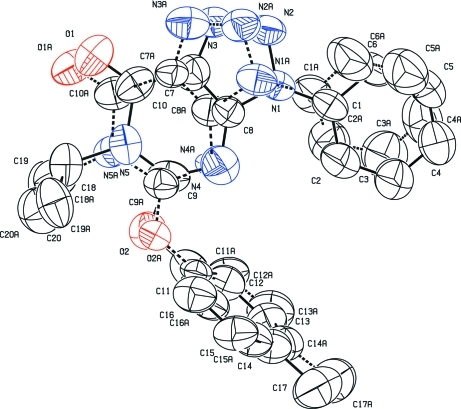
The molecular structure of (I), showing the atom-labelling scheme. Dashed lines indicate the minor component of disorder. H atoms have been omitted and displacement parameters are drawn at the 50% probability level.

## supplementary crystallographic information

### Comment

The derivatives of heterocycles containing the 8-azaguanine system, which are well known bioisosteres of guanine, are of great importance because of their remarkable biological properties. Some of these activities include antimicrobial or antifungal activities (Roblin *et al.*, 1945; Ding *et al.*, 2004), encephaloma cell inhibitor activity (Mitchell *et al.*, 1950; Levine *et al.*, 1963), antileukemie activity (Montgomery *et al.*, 1962), hypersusceptibility inhibitor activity and acesodyne activity (Yamamoto *et al.*, 1967; Bariana, 1971; Holland *et al.*, 1975).

In recent years, we have been engaged in the preparation of the derivatives of 8-azaguanine *via* aza-Wittig reaction of beta-ethoxycarbonyl iminophosphorane with aromatic isocyanate (Zhao, Xie *et al.*, 2005). As a continuation of our research for new biologically active heterocycles, the title compound, (I), was obtained from beta-ethoxycarbonyl iminophosphorane and alphalic isocyanate, and the crystal structure is reported herein.

The molecules of (I), which lie in general positions, exhibit 'whole molecule disorder' with the site-occupancy factors of 0.57 (2) and 0.43 (2), (Fig.1). The bond lengths and angles within the triazolopyrimidinone moiety are in good agreement with those observed for closely related structures. As reported for related compounds (Ferguson *et al.*, 1998; Maldonado *et al.*, 2006; Zeng, Deng *et al.*, 2009; Zeng, Liu *et al.*, 2009; Zhao, Hu *et al.*, 2005; Zhao, Wang & Ding, 2005; Wang *et al.*, 2006, 2008; Xiao *et al.*, 2007; Chen & Shi, 2006), all ring atoms in the pyrimidine ring system are essentially coplanar (maximum deviation -0.033 (17) Å for atom N4), indicating that the moiety is a conjugate system.

There are no inter- or intra- molecular hydrogen bonding interactions. The molecular conformation and crystal packing are stabilized by π–π stacking interactions occurring between symmetry realted triazole and pyrimidine rings, with centroid-to-centroid distances of 3.594 (10) Å.

### Experimental

To a solution of carbodiimide in CH_2_Cl_2_/CH_3_CN (1:4 *v*/*v*, 15 ml) prepared according to the literature method (Zeng *et al.*, 2006), was added *p*-cresol (3 mmol) and excess K_2_CO_3_. After the reaction mixture was stirred for 12 h. The solvent was removed under reduced pressure and the residue was recrystallized from EtOH to give the title compound (I) in yield of 85% (m.p. 436 K). Elemental analysis: calculated for C_20_H_19_N_5_O_2_: C, 66.47; H, 5.30; N, 19.38%. Found: C, 65.52; H, 5.63; N, 18.89%. Crystals suitable for singlecrystal X-ray diffraction were obtained from hexane and dichloromethane (1:3 *v*/*v*) at room temperature.

### Refinement

In the absense of significant anomalous dispersion effects Friedel pairs were merged. An examination of the data using PLATON (Spek, 2009) indicated that the crystal was not twinned. H atoms were placed at calculated positions and treated as riding atoms, with C—H = 0.93–0.98 Å, and *U*_iso_(H) = 1.2*U*_eq_(C) for CH or 1.5*U*_eq_(C) for CH_3_.

### Crystal data

**Table:**

C_20_H_19_N_5_O_2_	*F*(000) = 1520
*M**_r_* = 361.40	*D*_x_ = 1.282 Mg m^−^^3^
Orthorhombic, *C*222_1_	Mo *K*α radiation, λ = 0.71073 Å
Hall symbol: C 2c 2	Cell parameters from 7338 reflections
*a* = 10.2335 (6) Å	θ = 2.2–22.6°
*b* = 21.8532 (12) Å	µ = 0.09 mm^−^^1^
*c* = 16.7441 (9) Å	*T* = 298 K
*V* = 3744.6 (4) Å^3^	Block, colorless
*Z* = 8	0.20 × 0.20 × 0.20 mm

### Data collection

**Table:**

Bruker SMART CCD diffractometer	1790 reflections with *I* > 2σ(*I*)
Radiation source: fine-focus sealed tube	*R*_int_ = 0.023
graphite	θ_max_ = 27.0°, θ_min_ = 2.2°
φ and ω scans	*h* = −13→13
21260 measured reflections	*k* = −27→27
2285 independent reflections	*l* = −21→21

### Refinement

**Table:**

Refinement on *F*^2^	Primary atom site location: structure-invariant direct methods
Least-squares matrix: full	Secondary atom site location: difference Fourier map
*R*[*F*^2^ > 2σ(*F*^2^)] = 0.050	Hydrogen site location: inferred from neighbouring sites
*wR*(*F*^2^) = 0.144	H-atom parameters constrained
*S* = 1.06	*w* = 1/[σ^2^(*F*_o_^2^) + (0.1088*P*)^2^ + 0.0578*P*] where *P* = (*F*_o_^2^ + 2*F*_c_^2^)/3
2285 reflections	(Δ/σ)_max_ < 0.001
446 parameters	Δρ_max_ = 0.17 e Å^−^^3^
15 restraints	Δρ_min_ = −0.17 e Å^−^^3^

### Special details

**Table:**

Geometry. All e.s.d.'s (except the e.s.d. in the dihedral angle between two l.s. planes) are estimated using the full covariance matrix. The cell e.s.d.'s are taken into account individually in the estimation of e.s.d.'s in distances, angles and torsion angles; correlations between e.s.d.'s in cell parameters are only used when they are defined by crystal symmetry. An approximate (isotropic) treatment of cell e.s.d.'s is used for estimating e.s.d.'s involving l.s. planes.
Refinement. Refinement of *F*^2^ against ALL reflections. The weighted *R*-factor *wR* and goodness of fit *S* are based on *F*^2^, conventional *R*-factors *R* are based on *F*, with *F* set to zero for negative *F*^2^. The threshold expression of *F*^2^ > σ(*F*^2^) is used only for calculating *R*-factors(gt) *etc*. and is not relevant to the choice of reflections for refinement. *R*-factors based on *F*^2^ are statistically about twice as large as those based on *F*, and *R*- factors based on ALL data will be even larger.

### Fractional atomic coordinates and isotropic or equivalent isotropic displacement parameters (Å^2^)

**Table:**

	*x*	*y*	*z*	*U*_iso_*/*U*_eq_	Occ. (<1)
O1	0.5967 (13)	0.0694 (5)	0.1729 (8)	0.111 (2)	0.57 (2)
O2	0.3920 (17)	0.2076 (8)	0.0153 (9)	0.096 (3)	0.57 (2)
N1	0.6946 (14)	0.2696 (5)	0.2174 (10)	0.069 (2)	0.57 (2)
N2	0.7566 (13)	0.2328 (6)	0.2727 (7)	0.082 (2)	0.57 (2)
N3	0.7249 (10)	0.1753 (5)	0.2602 (6)	0.082 (3)	0.57 (2)
N4	0.5505 (14)	0.2489 (7)	0.1072 (8)	0.103 (5)	0.57 (2)
N5	0.500 (2)	0.1435 (10)	0.0927 (11)	0.085 (3)	0.57 (2)
C1	0.7134 (7)	0.3323 (3)	0.2161 (3)	0.0691 (17)	0.57 (2)
C2	0.6409 (8)	0.3694 (3)	0.1653 (6)	0.105 (3)	0.57 (2)
H2	0.5805	0.3521	0.1305	0.126*	0.57 (2)
C3	0.6587 (8)	0.4325 (3)	0.1665 (7)	0.117 (3)	0.57 (2)
H3	0.6102	0.4573	0.1325	0.141*	0.57 (2)
C4	0.7490 (7)	0.4584 (4)	0.2184 (5)	0.113 (3)	0.57 (2)
H4	0.7609	0.5006	0.2192	0.135*	0.57 (2)
C5	0.8215 (8)	0.4213 (5)	0.2692 (3)	0.110 (3)	0.57 (2)
H5	0.8819	0.4386	0.3040	0.132*	0.57 (2)
C6	0.8037 (8)	0.3582 (5)	0.2681 (3)	0.102 (3)	0.57 (2)
H6	0.8522	0.3334	0.3021	0.123*	0.57 (2)
C7	0.6443 (19)	0.1776 (9)	0.1971 (12)	0.081 (3)	0.57 (2)
C8	0.6235 (13)	0.2323 (6)	0.1690 (7)	0.062 (2)	0.57 (2)
C9	0.489 (3)	0.2026 (13)	0.0766 (15)	0.090 (5)	0.57 (2)
C10	0.586 (2)	0.1249 (10)	0.1598 (12)	0.085 (4)	0.57 (2)
C11	0.3987 (9)	0.2670 (3)	−0.0196 (6)	0.104 (4)	0.57 (2)
C12	0.3310 (9)	0.3155 (4)	0.0145 (5)	0.082 (3)	0.57 (2)
H12	0.2821	0.3095	0.0606	0.099*	0.57 (2)
C13	0.3364 (9)	0.3732 (3)	−0.0204 (6)	0.090 (2)	0.57 (2)
H13	0.2911	0.4057	0.0023	0.108*	0.57 (2)
C14	0.4095 (8)	0.3823 (4)	−0.0894 (6)	0.084 (2)	0.57 (2)
C15	0.4772 (9)	0.3337 (6)	−0.1235 (5)	0.097 (3)	0.57 (2)
H15	0.5261	0.3398	−0.1696	0.116*	0.57 (2)
C16	0.4718 (10)	0.2760 (5)	−0.0886 (6)	0.095 (2)	0.57 (2)
H16	0.5171	0.2435	−0.1113	0.114*	0.57 (2)
C17	0.4124 (14)	0.4466 (7)	−0.1241 (10)	0.136 (4)	0.57 (2)
H17A	0.3397	0.4696	−0.1038	0.204*	0.57 (2)
H17B	0.4069	0.4445	−0.1812	0.204*	0.57 (2)
H17C	0.4925	0.4664	−0.1090	0.204*	0.57 (2)
C18	0.4324 (15)	0.0923 (7)	0.0447 (9)	0.095 (3)	0.57 (2)
H18	0.4657	0.0539	0.0670	0.114*	0.57 (2)
C19	0.2897 (15)	0.0907 (8)	0.0583 (10)	0.125 (5)	0.57 (2)
H19A	0.2496	0.1250	0.0320	0.188*	0.57 (2)
H19B	0.2723	0.0928	0.1145	0.188*	0.57 (2)
H19C	0.2545	0.0534	0.0371	0.188*	0.57 (2)
C20	0.4717 (15)	0.0914 (10)	−0.0428 (10)	0.112 (3)	0.57 (2)
H20A	0.4440	0.0536	−0.0666	0.167*	0.57 (2)
H20B	0.5650	0.0950	−0.0470	0.167*	0.57 (2)
H20C	0.4311	0.1251	−0.0700	0.167*	0.57 (2)
C1A	0.7079 (12)	0.3091 (7)	0.2237 (6)	0.094 (3)	0.43 (2)
C2A	0.6177 (10)	0.3523 (7)	0.1975 (9)	0.111 (4)	0.43 (2)
H2A	0.5398	0.3396	0.1742	0.134*	0.43 (2)
C3A	0.6440 (10)	0.4143 (7)	0.2062 (12)	0.142 (5)	0.43 (2)
H3A	0.5837	0.4432	0.1887	0.170*	0.43 (2)
C4A	0.7605 (13)	0.4332 (8)	0.2410 (9)	0.134 (5)	0.43 (2)
H4A	0.7781	0.4748	0.2468	0.161*	0.43 (2)
C5A	0.8507 (13)	0.3901 (10)	0.2672 (6)	0.140 (6)	0.43 (2)
H5A	0.9286	0.4028	0.2905	0.168*	0.43 (2)
C6A	0.8244 (12)	0.3281 (10)	0.2585 (7)	0.136 (5)	0.43 (2)
H6A	0.8848	0.2992	0.2761	0.163*	0.43 (2)
C7A	0.6291 (16)	0.1569 (9)	0.1872 (9)	0.060 (3)	0.43 (2)
C8A	0.610 (2)	0.2128 (9)	0.1634 (11)	0.070 (4)	0.43 (2)
C9A	0.480 (3)	0.1980 (13)	0.0641 (17)	0.067 (4)	0.43 (2)
C10A	0.559 (3)	0.1085 (15)	0.1430 (15)	0.081 (4)	0.43 (2)
C11A	0.4049 (8)	0.2736 (4)	−0.0202 (5)	0.056 (2)	0.43 (2)
C12A	0.3332 (13)	0.3137 (5)	0.0270 (7)	0.102 (5)	0.43 (2)
H12A	0.2928	0.2996	0.0732	0.122*	0.43 (2)
C13A	0.3218 (16)	0.3748 (5)	0.0050 (10)	0.122 (5)	0.43 (2)
H13A	0.2738	0.4016	0.0366	0.147*	0.43 (2)
C14A	0.3822 (16)	0.3958 (4)	−0.0641 (10)	0.094 (4)	0.43 (2)
C15A	0.4539 (15)	0.3557 (7)	−0.1113 (6)	0.089 (4)	0.43 (2)
H15A	0.4943	0.3698	−0.1576	0.107*	0.43 (2)
C16A	0.4653 (11)	0.2946 (6)	−0.0894 (5)	0.093 (3)	0.43 (2)
H16A	0.5133	0.2678	−0.1210	0.111*	0.43 (2)
C17A	0.377 (2)	0.4628 (10)	−0.0866 (14)	0.147 (6)	0.43 (2)
H17D	0.3001	0.4811	−0.0642	0.221*	0.43 (2)
H17E	0.3749	0.4667	−0.1437	0.221*	0.43 (2)
H17F	0.4532	0.4832	−0.0662	0.221*	0.43 (2)
C18A	0.3959 (18)	0.0886 (12)	0.0309 (13)	0.106 (5)	0.43 (2)
H18A	0.4062	0.0485	0.0563	0.127*	0.43 (2)
C19A	0.2578 (17)	0.1054 (11)	0.0417 (11)	0.107 (4)	0.43 (2)
H19D	0.2372	0.1396	0.0081	0.160*	0.43 (2)
H19E	0.2427	0.1162	0.0965	0.160*	0.43 (2)
H19F	0.2034	0.0713	0.0276	0.160*	0.43 (2)
C20A	0.441 (3)	0.0808 (14)	−0.0529 (15)	0.134 (8)	0.43 (2)
H20D	0.3923	0.0485	−0.0778	0.200*	0.43 (2)
H20E	0.5323	0.0708	−0.0532	0.200*	0.43 (2)
H20F	0.4274	0.1183	−0.0818	0.200*	0.43 (2)
O1A	0.5643 (15)	0.0529 (8)	0.1518 (9)	0.105 (3)	0.43 (2)
O2A	0.4187 (19)	0.2151 (8)	0.0014 (9)	0.076 (3)	0.43 (2)
N1A	0.686 (2)	0.2462 (10)	0.2155 (15)	0.080 (4)	0.43 (2)
N2A	0.7450 (14)	0.2046 (8)	0.2667 (8)	0.086 (5)	0.43 (2)
N3A	0.7055 (19)	0.1495 (10)	0.2479 (11)	0.094 (4)	0.43 (2)
N4A	0.5407 (11)	0.2413 (7)	0.1048 (5)	0.057 (2)	0.43 (2)
N5A	0.479 (2)	0.1328 (10)	0.0799 (11)	0.074 (4)	0.43 (2)

### Atomic displacement parameters (Å^2^)

**Table:**

	*U*^11^	*U*^22^	*U*^33^	*U*^12^	*U*^13^	*U*^23^
O1	0.131 (6)	0.082 (4)	0.120 (6)	−0.009 (3)	−0.002 (4)	0.020 (4)
O2	0.090 (5)	0.086 (5)	0.111 (7)	−0.007 (3)	−0.014 (4)	0.009 (4)
N1	0.066 (3)	0.087 (7)	0.055 (2)	−0.002 (6)	−0.0045 (19)	0.004 (7)
N2	0.091 (3)	0.094 (6)	0.061 (2)	0.008 (5)	−0.014 (2)	0.006 (4)
N3	0.097 (7)	0.085 (10)	0.064 (5)	−0.003 (8)	−0.011 (4)	0.016 (8)
N4	0.111 (8)	0.096 (6)	0.103 (7)	0.004 (5)	−0.015 (5)	−0.008 (4)
N5	0.088 (5)	0.077 (5)	0.091 (6)	−0.010 (3)	0.001 (4)	−0.014 (4)
C1	0.063 (2)	0.092 (4)	0.052 (2)	0.000 (3)	0.000 (2)	−0.007 (2)
C2	0.103 (5)	0.108 (5)	0.104 (5)	0.001 (3)	−0.034 (4)	−0.005 (4)
C3	0.128 (5)	0.094 (4)	0.129 (7)	0.010 (4)	−0.028 (5)	−0.012 (4)
C4	0.140 (6)	0.096 (4)	0.102 (5)	0.003 (4)	−0.006 (4)	−0.028 (4)
C5	0.131 (6)	0.114 (6)	0.086 (4)	−0.008 (5)	−0.028 (4)	−0.027 (4)
C6	0.122 (6)	0.115 (7)	0.070 (3)	−0.014 (5)	−0.031 (3)	−0.003 (3)
C7	0.086 (5)	0.079 (10)	0.079 (5)	−0.004 (5)	0.003 (4)	0.002 (5)
C8	0.060 (3)	0.070 (5)	0.056 (4)	−0.002 (4)	0.002 (3)	0.000 (4)
C9	0.084 (6)	0.109 (11)	0.077 (9)	0.020 (7)	−0.009 (6)	0.007 (6)
C10	0.084 (9)	0.082 (12)	0.089 (12)	0.003 (6)	0.013 (7)	0.018 (7)
C11	0.093 (7)	0.119 (9)	0.101 (9)	−0.009 (6)	−0.017 (6)	−0.010 (7)
C12	0.075 (5)	0.095 (7)	0.077 (3)	0.002 (4)	−0.002 (3)	−0.001 (3)
C13	0.077 (4)	0.090 (5)	0.104 (6)	0.019 (3)	−0.004 (4)	0.001 (3)
C14	0.071 (4)	0.097 (5)	0.083 (5)	0.009 (4)	−0.003 (3)	0.004 (4)
C15	0.092 (4)	0.112 (7)	0.086 (4)	0.025 (5)	−0.002 (3)	0.009 (5)
C16	0.090 (4)	0.112 (5)	0.083 (4)	0.025 (4)	0.000 (3)	−0.004 (3)
C17	0.129 (7)	0.128 (8)	0.152 (10)	0.006 (5)	−0.005 (7)	0.040 (7)
C18	0.102 (6)	0.075 (4)	0.106 (6)	−0.008 (4)	0.005 (5)	−0.016 (4)
C19	0.121 (9)	0.114 (7)	0.140 (9)	−0.031 (7)	0.000 (7)	−0.016 (6)
C20	0.111 (6)	0.113 (6)	0.111 (6)	−0.006 (5)	0.015 (5)	−0.052 (4)
C1A	0.098 (6)	0.115 (10)	0.071 (5)	−0.016 (9)	0.006 (4)	0.007 (8)
C2A	0.110 (6)	0.112 (7)	0.113 (8)	0.008 (6)	0.001 (6)	−0.018 (6)
C3A	0.148 (10)	0.133 (9)	0.145 (12)	0.011 (8)	−0.008 (9)	−0.010 (9)
C4A	0.166 (14)	0.131 (10)	0.104 (9)	−0.021 (9)	0.015 (9)	−0.039 (10)
C5A	0.165 (14)	0.154 (16)	0.102 (7)	−0.014 (11)	−0.009 (7)	−0.015 (9)
C6A	0.156 (11)	0.162 (13)	0.090 (6)	−0.024 (9)	−0.019 (6)	−0.010 (8)
C7A	0.065 (6)	0.068 (12)	0.049 (5)	0.000 (6)	0.000 (4)	0.008 (6)
C8A	0.071 (5)	0.081 (10)	0.058 (4)	0.003 (7)	0.003 (4)	0.002 (7)
C9A	0.070 (8)	0.075 (7)	0.056 (6)	0.013 (5)	−0.001 (4)	−0.006 (4)
C10A	0.083 (10)	0.094 (16)	0.067 (7)	0.010 (8)	0.011 (6)	0.014 (7)
C11A	0.055 (4)	0.064 (4)	0.049 (4)	0.012 (3)	−0.021 (3)	−0.001 (3)
C12A	0.097 (10)	0.107 (11)	0.101 (7)	0.022 (8)	−0.004 (6)	0.015 (6)
C13A	0.109 (8)	0.137 (11)	0.121 (10)	0.013 (7)	−0.006 (7)	0.005 (7)
C14A	0.085 (8)	0.101 (7)	0.096 (10)	0.011 (5)	−0.016 (6)	0.000 (6)
C15A	0.090 (8)	0.103 (9)	0.074 (5)	0.005 (7)	−0.003 (4)	0.000 (6)
C16A	0.094 (6)	0.112 (8)	0.072 (6)	0.014 (5)	−0.018 (5)	−0.014 (5)
C17A	0.154 (14)	0.129 (11)	0.159 (15)	−0.003 (9)	−0.017 (11)	0.024 (10)
C18A	0.104 (11)	0.111 (10)	0.103 (8)	0.005 (8)	0.003 (7)	0.014 (7)
C19A	0.093 (7)	0.131 (11)	0.096 (7)	0.005 (6)	0.011 (6)	−0.011 (6)
C20A	0.150 (16)	0.133 (15)	0.118 (10)	0.004 (11)	−0.006 (10)	−0.020 (9)
O1A	0.121 (7)	0.090 (7)	0.106 (6)	0.010 (5)	−0.006 (5)	0.028 (5)
O2A	0.092 (8)	0.068 (4)	0.069 (4)	−0.005 (4)	−0.025 (5)	0.002 (4)
N1A	0.080 (6)	0.105 (12)	0.056 (4)	0.008 (8)	0.000 (4)	0.004 (9)
N2A	0.097 (8)	0.091 (15)	0.069 (6)	−0.003 (13)	−0.004 (5)	0.015 (13)
N3A	0.094 (6)	0.116 (11)	0.072 (5)	0.006 (8)	−0.005 (4)	0.016 (7)
N4A	0.048 (3)	0.076 (5)	0.046 (4)	0.006 (3)	−0.012 (3)	0.001 (3)
N5A	0.087 (8)	0.071 (9)	0.063 (5)	0.011 (6)	0.001 (5)	0.005 (5)

### Geometric parameters (Å, °)

**Table:**

O1—C10	1.24 (3)	C1A—C2A	1.3900
O2—C11	1.425 (17)	C1A—C6A	1.3900
O2—C9	1.43 (2)	C1A—N1A	1.401 (15)
N1—C8	1.360 (15)	C2A—C3A	1.3900
N1—N2	1.381 (19)	C2A—H2A	0.9300
N1—C1	1.383 (9)	C3A—C4A	1.3900
N2—N3	1.315 (9)	C3A—H3A	0.9300
N3—C7	1.34 (3)	C4A—C5A	1.3900
N4—C9	1.300 (13)	C4A—H4A	0.9300
N4—C8	1.328 (11)	C5A—C6A	1.3900
N5—C9	1.32 (3)	C5A—H5A	0.9300
N5—C10	1.48 (3)	C6A—H6A	0.9300
N5—C18	1.543 (11)	C7A—N3A	1.29 (3)
C1—C2	1.3900	C7A—C8A	1.30 (2)
C1—C6	1.3900	C7A—C10A	1.477 (16)
C2—C3	1.3900	C8A—N4A	1.358 (13)
C2—H2	0.9300	C8A—N1A	1.38 (2)
C3—C4	1.3900	C9A—O2A	1.28 (3)
C3—H3	0.9300	C9A—N4A	1.321 (12)
C4—C5	1.3900	C9A—N5A	1.45 (3)
C4—H4	0.9300	C10A—O1A	1.23 (4)
C5—C6	1.3900	C10A—N5A	1.44 (4)
C5—H5	0.9300	C11A—O2A	1.34 (2)
C6—H6	0.9300	C11A—C12A	1.3900
C7—C8	1.30 (2)	C11A—C16A	1.3900
C7—C10	1.440 (13)	C12A—C13A	1.3900
C11—C12	1.3900	C12A—H12A	0.9300
C11—C16	1.3900	C13A—C14A	1.3900
C12—C13	1.3900	C13A—H13A	0.9300
C12—H12	0.9300	C14A—C15A	1.3900
C13—C14	1.3900	C14A—C17A	1.51 (2)
C13—H13	0.9300	C15A—C16A	1.3900
C14—C15	1.3900	C15A—H15A	0.9300
C14—C17	1.522 (14)	C16A—H16A	0.9300
C15—C16	1.3900	C17A—H17D	0.9600
C15—H15	0.9300	C17A—H17E	0.9600
C16—H16	0.9300	C17A—H17F	0.9600
C17—H17A	0.9600	C18A—C19A	1.471 (14)
C17—H17B	0.9600	C18A—C20A	1.487 (14)
C17—H17C	0.9600	C18A—N5A	1.528 (15)
C18—C19	1.478 (12)	C18A—H18A	0.9800
C18—C20	1.519 (12)	C19A—H19D	0.9600
C18—H18	0.9800	C19A—H19E	0.9600
C19—H19A	0.9600	C19A—H19F	0.9600
C19—H19B	0.9600	C20A—H20D	0.9600
C19—H19C	0.9600	C20A—H20E	0.9600
C20—H20A	0.9600	C20A—H20F	0.9600
C20—H20B	0.9600	N1A—N2A	1.39 (3)
C20—H20C	0.9600	N2A—N3A	1.309 (14)
			
C11—O2—C9	109.3 (16)	C3A—C4A—C5A	120.0
C8—N1—N2	107.2 (9)	C3A—C4A—H4A	120.0
C8—N1—C1	131.2 (12)	C5A—C4A—H4A	120.0
N2—N1—C1	121.6 (11)	C6A—C5A—C4A	120.0
N3—N2—N1	109.6 (11)	C6A—C5A—H5A	120.0
N2—N3—C7	104.1 (12)	C4A—C5A—H5A	120.0
C9—N4—C8	111.6 (12)	C5A—C6A—C1A	120.0
C9—N5—C10	118.4 (13)	C5A—C6A—H6A	120.0
C9—N5—C18	124.0 (17)	C1A—C6A—H6A	120.0
C10—N5—C18	117.6 (17)	N3A—C7A—C8A	116.8 (15)
N1—C1—C2	120.9 (7)	N3A—C7A—C10A	126.7 (17)
N1—C1—C6	119.1 (7)	C8A—C7A—C10A	116.4 (18)
C2—C1—C6	120.0	C7A—C8A—N4A	136.9 (15)
C3—C2—C1	120.0	C7A—C8A—N1A	102.5 (14)
C3—C2—H2	120.0	N4A—C8A—N1A	120.6 (16)
C1—C2—H2	120.0	O2A—C9A—N4A	116 (2)
C4—C3—C2	120.0	O2A—C9A—N5A	115.8 (16)
C4—C3—H3	120.0	N4A—C9A—N5A	127.8 (19)
C2—C3—H3	120.0	O1A—C10A—N5A	118.8 (15)
C5—C4—C3	120.0	O1A—C10A—C7A	129 (2)
C5—C4—H4	120.0	N5A—C10A—C7A	112 (2)
C3—C4—H4	120.0	O2A—C11A—C12A	120.3 (9)
C4—C5—C6	120.0	O2A—C11A—C16A	119.7 (9)
C4—C5—H5	120.0	C12A—C11A—C16A	120.0
C6—C5—H5	120.0	C11A—C12A—C13A	120.0
C5—C6—C1	120.0	C11A—C12A—H12A	120.0
C5—C6—H6	120.0	C13A—C12A—H12A	120.0
C1—C6—H6	120.0	C14A—C13A—C12A	120.0
C8—C7—N3	114.7 (9)	C14A—C13A—H13A	120.0
C8—C7—C10	120.5 (16)	C12A—C13A—H13A	120.0
N3—C7—C10	124.7 (17)	C15A—C14A—C13A	120.0
C7—C8—N4	128.6 (12)	C15A—C14A—C17A	119.1 (10)
C7—C8—N1	104.4 (11)	C13A—C14A—C17A	120.8 (10)
N4—C8—N1	127.0 (13)	C14A—C15A—C16A	120.0
N4—C9—N5	129.5 (18)	C14A—C15A—H15A	120.0
N4—C9—O2	124 (2)	C16A—C15A—H15A	120.0
N5—C9—O2	106.5 (16)	C15A—C16A—C11A	120.0
O1—C10—C7	131.8 (17)	C15A—C16A—H16A	120.0
O1—C10—N5	117.2 (13)	C11A—C16A—H16A	120.0
C7—C10—N5	110.9 (16)	C14A—C17A—H17D	109.5
C12—C11—C16	120.0	C14A—C17A—H17E	109.5
C12—C11—O2	120.2 (8)	H17D—C17A—H17E	109.5
C16—C11—O2	119.8 (8)	C14A—C17A—H17F	109.5
C13—C12—C11	120.0	H17D—C17A—H17F	109.5
C13—C12—H12	120.0	H17E—C17A—H17F	109.5
C11—C12—H12	120.0	C19A—C18A—C20A	116.2 (14)
C12—C13—C14	120.0	C19A—C18A—N5A	108.2 (19)
C12—C13—H13	120.0	C20A—C18A—N5A	113.9 (17)
C14—C13—H13	120.0	C19A—C18A—H18A	105.9
C15—C14—C13	120.0	C20A—C18A—H18A	105.9
C15—C14—C17	122.7 (7)	N5A—C18A—H18A	105.9
C13—C14—C17	117.3 (7)	C18A—C19A—H19D	109.5
C14—C15—C16	120.0	C18A—C19A—H19E	109.5
C14—C15—H15	120.0	H19D—C19A—H19E	109.5
C16—C15—H15	120.0	C18A—C19A—H19F	109.5
C15—C16—C11	120.0	H19D—C19A—H19F	109.5
C15—C16—H16	120.0	H19E—C19A—H19F	109.5
C11—C16—H16	120.0	C18A—C20A—H20D	109.5
C19—C18—C20	114.2 (11)	C18A—C20A—H20E	109.5
C19—C18—N5	112.4 (12)	H20D—C20A—H20E	109.5
C20—C18—N5	113.1 (13)	C18A—C20A—H20F	109.5
C19—C18—H18	105.4	H20D—C20A—H20F	109.5
C20—C18—H18	105.4	H20E—C20A—H20F	109.5
N5—C18—H18	105.4	C9A—O2A—C11A	123.7 (16)
C2A—C1A—C6A	120.0	C8A—N1A—N2A	107.0 (11)
C2A—C1A—N1A	121.8 (13)	C8A—N1A—C1A	132 (2)
C6A—C1A—N1A	118.2 (13)	N2A—N1A—C1A	121 (2)
C3A—C2A—C1A	120.0	N3A—N2A—N1A	108.5 (17)
C3A—C2A—H2A	120.0	C7A—N3A—N2A	105.1 (19)
C1A—C2A—H2A	120.0	C9A—N4A—C8A	106.8 (13)
C2A—C3A—C4A	120.0	C10A—N5A—C9A	119.7 (16)
C2A—C3A—H3A	120.0	C10A—N5A—C18A	118.5 (17)
C4A—C3A—H3A	120.0	C9A—N5A—C18A	121.8 (18)
